# Colliding Epidemics: Research Gaps and Implementation Science Opportunities for Tobacco Use and HIV/AIDS in Low- and Middle-Income Countries

**DOI:** 10.1155/2022/6835146

**Published:** 2022-06-17

**Authors:** Mark Parascandola, Gila Neta, Michele Bloch, Satish Gopal

**Affiliations:** ^1^Center for Global Health, National Cancer Institute, Bethesda, Maryland, USA; ^2^Office of the Director, Division of Cancer Control and Population Sciences, National Cancer Institute, Bethesda, Maryland, USA; ^3^Tobacco Control Research Branch, Behavioral Research Program, Division of Cancer Control and Population Sciences, National Cancer Institute, Bethesda, Maryland, USA

## Abstract

**Introduction:**

Tobacco use is a leading cause of cancer death among people living with HIV (PLWH) worldwide, and smoking prevalence tends to be higher among PLWH. The burden of both HIV/AIDS and tobacco use is increasingly concentrated in low- and middle-income countries (LMICs), where resources to address these challenges are often limited. However, there has been limited effort to date to integrate tobacco cessation into HIV programs in LMICs.

**Methods:**

We searched the literature (searching was conducted between October 1 and December 31, 2020) using PubMed including search terms “tobacco” and “HIV” and “cessation” over the past ten years (searching for articles published between December 1, 2010, and December 1, 2020) to identify original research studies on tobacco cessation interventions conducted in LMICs for PLWH. We also conducted an analysis of NCI-funded research grants on tobacco cessation and HIV awarded during fiscal years 2010 to 2020. *Results and Discussion*. Existing evidence suggests that conventional tobacco cessation treatments may be less effective among PLWH. Moreover, while substantial evidence exists to support a range of cessation interventions, most of this evidence comes from HICs and is only partly applicable to the evolving social, economic, and cultural climate of many LMICs. There is an urgent need to develop, adapt, and implement effective tobacco control and cessation interventions targeted to PLWH in LMICs, as well as to generate evidence from these settings. Implementation science provides tools develop and test strategies to overcome barriers and to integrate and scale up cessation services within existing HIV treatment settings.

**Conclusion:**

There is a unique opportunity to address HIV and tobacco use in a coordinated way in LMICs by integrating evidence-based tobacco cessation into HIV programs.

## 1. Introduction

The intersection between tobacco use and the global HIV/AIDS epidemic poses a serious and underappreciated public health threat in many parts of the world. Multination surveys have shown that smoking prevalence tends to be higher among persons living with HIV (PLWH) compared with their HIV-negative counterparts across regions and country income categories [1, 2]. The burden of both HIV/AIDS and tobacco use is increasingly concentrated in low- and middle-income countries (LMICs), where resources to address these challenges are often limited. To date, however, these intersecting epidemics have not been addressed in a coordinated way.

Limited data exist on the full extent of the combined burden of tobacco and HIV in LMICs, the mechanisms through which tobacco use impacts HIV outcomes and the effectiveness of tailored interventions for tobacco cessation among PLWH in low-resource settings. Thus, there is an urgent need to develop, adapt, and implement effective tobacco control and cessation interventions targeted to PLWH. As we will describe, addressing the interaction between these two public health challenges in a coordinated manner could lead to meaningful advances in global health. Additionally, this paper will identify research opportunities grounded in implementation science to advance interventions for tobacco cessation among PLWH in LMICs.

## 2. Methods

We searched the literature (searching conducted between October 1 and December 31, 2020) using PubMed including search terms “tobacco” and “HIV” and “cessation” over the past ten years (studies published between December 1, 2010, and December 1, 2020) to identify original research studies on tobacco cessation interventions conducted in LMICs for PLWH. We also conducted an analysis of NCI-funded research grants on tobacco cessation and HIV awarded during fiscal years 2010 to 2020 and identified related publications. Additionally, UNAIDS and WHO HIV/AIDS and tuberculosis guidance documents from 2020 or the most recent available year were reviewed for mention of tobacco use and cessation.

### 2.1. The Colliding Epidemics of Tobacco and HIV

In 2019, there were 36.2 million adults (15+ years old) living with HIV (PLWH), the majority of them in LMICs [3]. Over half of PWLH (54%) live in Eastern and Southern Africa [4]. Characteristics of PLWH differ substantially across regions. For example, in the WHO Asia Pacific region, new infections are two times higher among men than among women and tend to be concentrated in specific subpopulations, including men who have sex with men, injection drug users, and sex workers and their clients. In contrast, in SSA, new infections are more common among women, especially adolescent girls and young women [5, 6].

Global investments in HIV/AIDS prevention and treatment have helped reduce morbidity and mortality from HIV [7]. AIDS-related deaths have been reduced by 60% since peaking in 2004 [8], and in that time, HIV prevention, care, and treatment programs have been established in many LMICs. Today, the majority of people living with HIV worldwide (67%, or 25.4 million) are receiving ART treatment [3]. By 2020, fourteen countries, including seven in SSA, had achieved the UNAIDS 90-90-90 targets for HIV diagnosis and treatment (targets that are yet to be achieved at a national level in the U.S.) [9]. In recent decades, multisector funding investments have built strong healthcare delivery systems and infrastructure for HIV treatment and prevention worldwide.

With the decline in infection-related mortality, noncommunicable diseases (NCDs) including cardiovascular diseases and malignancies have become major comorbidities and causes of death in HIV-infected populations [10]. Studies from the U.S. find that tobacco use accounts for one fifth of the cancer burden among PLWH [11], and that PLWH who adhere to ART but smoke is more likely to die from lung cancer than from AIDS-related causes [12, 13]. In fact, in the U.S., PLWH now lose more life-years to tobacco use than to HIV infection [14]. Thus, while advances in HIV treatment have contributed to substantially longer lifespans in PLWH, many LMICs are now facing a “double burden” of disease, with a rise in the prevalence of NCDs, such as cancer and heart disease, due to economic and lifestyle changes while still facing the continued burden of chronic infectious diseases such as HIV [15]. A recent systematic review highlights the lack of surveillance for NCDs among PLWH in LMICs and notes that, if left unaddressed, NCDs may undermine the effectiveness of global HIV/AIDS programs [16, 17].

There are over 1.3 billion tobacco users worldwide, of whom more than three quarters are male. In 2015, global prevalence of current tobacco use (including cigarettes as well as smokeless tobacco and other products) was 24.9% (40.3% among men; 9.5% among women). Over the past several decades, progress has been made in reducing tobacco use through implementation of evidence-based programs and policies. However, this progress has been primarily limited to high-income countries (HICs). Currently, over 84% of the world's tobacco users are in LMICs, where the future burden of tobacco related morbidity and mortality will be concentrated [18]. In fact, a 2018 World Health Organization (WHO) report projects that smoking prevalence will increase in Sub-Saharan Africa and the Eastern Mediterranean regions due to demographic changes, economic development, and tobacco product marketing [19].

### 2.2. Tobacco, HIV, and Comorbidities

A range of studies, largely from HICs, have documented that PLWH who smoke tobacco products experience greater morbidity and mortality than their nonsmoking counterparts [14, 20]. PLWH who smoke have increased risks of developing lung and other forms of cancer and are more likely to develop pneumonia, chronic obstructive pulmonary disease (COPD), and cardiovascular disease [21, 22]. For example, in a large cohort study involving 18,000 HIV-infected persons on ART from Europe and North America, smokers (who comprised 60% of the sample) had twice the mortality rate of nonsmokers, mostly from non-AIDS-related malignancies and cardiovascular disease [23]. Indeed, among patients on antiretroviral therapy (ART), tobacco use may account for 25% of total mortality [24].

There is also some evidence suggesting that tobacco smoking increases the risk of HIV/AIDS infection and disease progression, although the underlying mechanisms are not well understood. Tobacco smoking appears to be an independent and important risk factor for contracting HIV [25, 26]. In addition, PLWH who smoke have a greater rate of progression from HIV infection to AIDS and have a poorer response to ART. In a longitudinal study of a large HIV-infected cohort, Feldman and coworkers found that smokers receiving ART had poorer immunologic responses and a greater risk of virologic rebound compared with nonsmokers [27]. Smoking is also associated with lower ART adherence in patients with AIDS who smoke compared to those who do not [28]. At the same time, one study found that PLWH who quit smoking by age 40 have a much lower risk of lung cancer mortality compared with those who continue to smoke, and there is ample evidence from the general population that quitting at any age is beneficial [13].

Both tobacco use and HIV also contribute to the global burden of tuberculosis (TB). According to the WHO, at least one-third of PLWH are infected with latent TB and TB is the leading cause of death among PLWH, particularly in LMICs [13]. Moreover, there is sufficient evidence to conclude that cigarette smoking increases risk of TB infection and associated mortality [29]. According to one estimate, 25% of global TB mortality may be attributable to tobacco use [30].

### 2.3. A Coordinated Response to Tobacco and HIV

Recently, commentators have called for the need to address the “syndemic” of tobacco and HIV, along with TB, in a coordinated way [31, 32]. However, existing global HIV and TB programs and guidelines often fail to address tobacco use. For example, WHO and UNAIDS guidelines for treatment and care of PLWH make no mention of the impact of tobacco use on treatment outcomes or quality of life. The UNAIDS Fast Track Strategy, which sets an ambitious goal to end the AIDS epidemic by 2030, acknowledges the need for a more holistic approach to addressing comorbidities and other health needs of PLWH, but does not specifically address tobacco use [33]. Similarly, tobacco interventions are not mentioned in the global STOP TB plan [34] or in WHO recommendations on TB in people living with HIV/AIDS [35]. The importance of recording of patients' tobacco use status is rarely noted. For example, the Global AIDS Monitoring Framework indicators do not include assessment of tobacco use [36]. This is despite a 2007 World Health Organization report recommending the inclusion of brief counseling and pharmacologic therapy for tobacco dependence in TB treatment programs [37].

The WHO Global Action Plan for the Prevention and Control of NCDs, endorsed by the World Health Assembly in 2013, sets a target of a 30% relative reduction in prevalence of current tobacco use by 2025 [38]. Although 182 countries are parties to the WHO FCTC as of 2021, the tobacco control measures mandated by the treaty have yet to be fully implemented [39]. The 2019 WHO Report on the Global Tobacco Epidemic [40] reports that 65% of the world's population, and 61% of those living in LMICs, are now covered by at least one MPOWER measure (not including monitoring) at the highest level of achievement; although this represents a substantial increase over the previous decade, much remains to be done. Article 14 of the FCTC states that each party to the treaty shall develop context-appropriate evidence-based guidelines for treatment of tobacco dependence. However, implementation of Article 14 has been challenging. While most countries that are parties to the FCTC report offering some form of cessation support, in practice, this is often very limited in scope as many LMICs lack the infrastructure needed to offer widespread cessation support to the majority of tobacco users [41]. Important barriers to implementing the WHO FCTC Article 14 guidelines include lack of adequate healthcare infrastructure, low political priority, and lack of funding [42].

There is a unique opportunity to address HIV, along with TB coinfection, and tobacco use in a coordinated way by integrating tobacco cessation into the HIV and TB treatment context. HIV and TB treatment both require repeated contact with the health system and exchanges with healthcare workers. These interactions can serve as opportunities to discuss and address tobacco use, including offering support for cessation. In LMICs with a high HIV/TB burden, through support from the Global Fund for AIDS, TB, and malaria and other global funders, there is already substantial infrastructure in place for HIV and TB programs, which could serve as a platform for other health interventions. Given recent WHO guidelines for HIV emphasize the need to integrate services for common comorbidities including cancer into HIV programs [43]. Integrating tobacco cessation into existing programs could greatly expand access to cessation support, particularly benefitting communities most impacted by HIV/AIDS and tobacco [44]. Such integration is also likely to bring economic benefits, including improved outcomes and reduced health care costs for HIV/AIDS and TB programs in already overburdened countries [45].

### 2.4. Research towards Effective Interventions

Despite the many reasons to pursue greater integration of tobacco control into HIV prevention and treatment programs, little research has been done to develop or evaluate interventions for tobacco dependence in this setting. Research, both basic and applied, has been an essential element driving progress in tobacco control in HICs over the past half century. But while a substantial body of evidence exists to support a range of cessation interventions for the general public [46, 47], most of this evidence comes from HICs and is only partly applicable to the evolving social, economic, and cultural climate of many LMICs, especially in the context of tobacco dependence treatment across countries with diverse health systems, tobacco use behaviors, patterns of dependence, and tobacco product markets [48]. In particular, tobacco cessation interventions face unique challenges in LMICs, given limited resources and access to medications as well as diverse cultural and social contexts. There is a need for interventions tailored to these environments, including the development of low-cost cessation interventions and integration of cessation services into health systems [49]. A recent systematic review found few studies of the effectiveness of behavioral and pharmacologic treatments for tobacco dependence conducted in LMICs [50]. Additionally, conventional tobacco cessation interventions may need to be adapted for PLWH, given different patient characteristics and challenges, as well as the different settings and contexts in which those patients might be seeking care.

There is a tremendous opportunity to build on the existing knowledge, experience, and infrastructure around ART delivery to advance tobacco cessation for PLWH in LMICs. However, as noted above, there are additional challenges to delivering tobacco cessation in LMICs and among PLWH. Implementation science has advanced successful HIV care delivery models in LMICs through the development and testing of strategies including the use of task shifting, decentralized care delivery, peer support groups, and leveraging community health workers. These are strategies that could also be adapted to overcome obstacles to delivering effective tobacco cessation interventions to PLWH in LMICs [51]. The primary challenges are in adapting effective, evidence-based tobacco cessation treatments for the context of PLWH in LMICs, which requires an understanding of the barriers and facilitators to implementing tailored interventions in these settings and devising strategies to address these barriers [52, 53]. Agyepong et al. [54] present a generic implementation framework to describe the stages or steps of implementation research to advance an intervention, moving from the preimplementation phase, through the process of implementation, to the postimplementation phase. Currently, evidence remains limited both for adapting interventions so that they are feasible, acceptable, and effective in LMIC settings as well as understanding the unique implementation challenges and strategies to deliver tobacco cessation in LMICs. Thus, there is a need for research throughout the implementation continuum. In the following section, we outline a series of research questions and opportunities along the continuum for tobacco cessation among PLWH in LMICs. Sample research questions and related methods and models are summarized in Table 1.

### 2.5. Preimplementation

Existing literature, largely from HICs, finds that smoking cessation in PLWH presents unique challenges and that tailored treatments may be required to address this context [55, 56]. A 2016 Cochrane review found low quality evidence that combined tobacco cessation interventions were effective in helping PLWH achieve short-term abstinence, but evidence for long-term impact (abstinence for six months or more) was lacking [57]. PLWH exhibit faster nicotine metabolism, which may partially explain lower response to nicotine replacement therapy [58]. Two recent studies have shown a positive effect on cessation with varenicline in PLWH, though abstinence rates tend to be lower than those seen in the general population of smokers [59, 60]. Other reviews have suggested that treatments tailored to PLWH, by addressing complex medical and psychosocial factors, may show greater success [61, 55, 62]. Adding text messaging and telephone counseling to pharmacotherapy may also increase adherence among patients with HIV [63]. However, research on interventions tailored to HIV-positive persons remains extremely limited [58].

In the context of PLWH in LMICs, research on tobacco cessation is extremely limited, though some formative work has been done to identify factors that may impact likelihood of quitting. A cross-sectional study of smoking behavior among PLWH in Rio de Janeiro, Brazil, identified higher prevalence of alcohol and illicit drug use, as well as a higher proportion of men who have sex with men, among current versus former smokers, suggesting cessation may be more challenging for these groups [64]. In a qualitative assessment following a randomized controlled trial for smoking cessation in PLWH in South Africa, participants reported economic stressors (poverty and unemployment) and lack of social support as key barriers to quitting, suggesting the need to address barriers at multiple levels [65]. In a quasiexperimental study to assess a behavioral cessation intervention for PLWH in Nepal, the authors found that a 1.5 hour informational session led to an increase in smoking-related knowledge and intention to quit [66]. Nguyen et al. found that among a sample of HIV-positive smokers in Vietnam, higher income and self-reported pain were associated with higher motivation to quit smoking, while taking methadone, binge drinking, and use of illicit drugs were associated with lower motivation to quit [67]. However, such short-term and cross-sectional studies do not provide evidence of sustained behavior change. Additionally, there is a lack of studies of cessation among PLWH who are users of noncigarette tobacco products, such as hookah or smokeless tobacco, despite the high prevalence of these products in some LMICs [68, 69].

Digital technologies (such as mobile phone-based tools) offer novel opportunities for increasing the reach of cessation services, especially for challenging or low-resource settings in LMICs. In recent years, cell phone penetration has increased to over 90% in LMICs, a figure that is higher than the global average [70]. There is now moderate evidence that automated text message-based smoking cessation interventions result in higher quit rates than minimal smoking cessation support [71]. Preliminary studies of mHealth interventions for cessation among PLWH have yielded mixed results [72, 73]. Additionally, there is a need for more studies conducted in LMICs. For example, Do et al. identified 108 studies evaluating eHealth and mHealth interventions for smoking cessation, almost all of which were conducted in high-income countries [74]. WHO's mTB Tobacco program provides a platform for an SMS text message-based cessation intervention for TB patients which could potentially be adapted for the HIV context as well [75]. During the COVID-19 pandemic, there has been increased interest in the potential for digital health tools to provide health services while reducing risk of disease transmission, and such tools are likely to see more use in the future [76].

### 2.6. Adaptation

As described above, while there is a strong evidence base supporting the effectiveness of a variety of tobacco cessation interventions, these may require adaptation or tailoring for a specific context, such as when applying interventions from HICs to LMICs or from non-HIV-specific settings to PLWH. More recently, however, the implementation science literature has highlighted the need for greater attention to the fit between interventions and their settings and the potential for ongoing optimization over time [77]. There are a variety of ways in which adaptation can occur, both planned and unplanned, and it is important to be able to document adaptations, including what is modified (including by whom, when, where, or how the intervention is delivered), the extent to which the modification impacts fidelity, the reasons for the modification, and contextual factors that led to the modification [78]. One systematic review reported on adaptations of evidence-based interventions in HIV/AIDS, mental health, substance abuse, and chronic illness; frequent reasons for adaptation included the need for cultural appropriateness, focusing on a new target population, and implementing in a new setting [79]. The concept of transferability is helpful in identifying factors to consider in transferring (and adapting) an evidence-based intervention from one context to another, including characteristics of the target population, the environment, the intervention itself, and the transfer process [80].

One potential strategy is to adapt interventions that have previously been tested in other challenging and/or low-resource settings. For example, pilot studies testing the integration of smoking cessation interventions into the TB treatment context in some LMICs have shown promise for reducing smoking and improving TB treatment outcomes in low-resource settings. Pilot studies of smoking cessation in the context of TB treatment have been conducted in Brazil [81], Malaysia [82], China [83], Indonesia [84], Bangladesh [85], and Nepal [86]. A randomized controlled trial in Pakistan found that behavioral support alone or in combination with bupropion is effective in promoting cessation in smokers with suspected TB [87]. Another randomized trial among TB patients in South Africa, most of whom had coinfection with HIV, showed that motivational interviewing delivered by lay health workers was twice as effective in promoting smoking cessation as brief advice to quit [88]. These studies have generally supported the acceptability, feasibility, and need for additional implementation research in this area.

### 2.7. Barriers and Facilitators

A new diagnosis of HIV/AIDS or TB is a health event that provides a “teachable moment” for tobacco use cessation [89, 90] and a point at which patients are likely to be highly motivated to take actions that will improve the efficacy of their ART regimens. The HIV care context can provide a supportive environment for cessation attempts. However, there are a number of barriers and complicating factors that compromise the success of smoking cessation. In many countries, including some with a high HIV/AIDS and TB burden, tobacco use remains high (above 20%) among health professionals themselves, which is a barrier to implementing cessation programs [91]. HIV care providers are primarily focused on managing complications of HIV infection, and assessing smoking status and providing tobacco dependence treatment may be seen as a comparatively low priority. HIV/AIDS and TB patients are more likely to be socioeconomically disadvantaged compared to the general smoker population, and this affects access to cessation and educational interventions [92]. Moreover, a diversity of tobacco use patterns, policy environments, and health care resources mean that local, context-specific data are needed, and guidelines must be tailored to the environment in which they are to be implemented [93]. Research is needed to characterize and understand these potential barriers, as well as strategies to address them. Tools such as the Consolidated Framework for Implementation Research (CFIR) and the Exploration, Preparation, Implementation, Sustainment (EPIS) framework are widely used to formulate such research questions [94, 95] including in LMIC settings [96], by identifying key determinants and processes that are likely to influence successful implementation of evidence-based interventions.

### 2.8. Strategies for Scale-Up and Integration

Integration of tobacco cessation services into the HIV treatment context can be seen as both a challenge and an opportunity. Integration of tobacco cessation guidelines and practices requires identifying the appropriate practitioners to deliver the services, providing additional training to those practitioners, and ensuring they have the necessary supports in place to make it easy for them to deliver the services as part of the workflow, especially given their burden in busy clinics with limited resources and where the primary focus is on HIV treatment. Additionally, scale-up may require broader leadership buy in, practice facilitation, or monitoring systems that require further resources. However, at the same time, the extensive networks of community health workers and clinics that support HIV programs in some LMICs provide a platform on which tobacco cessation services could be scaled up to impact populations that might otherwise be difficult to reach.

To date, little research exists as to appropriate methods for effective integration of tobacco control interventions within HIV programs. Nor is there any information on the effect that regular, frequent contact with a health care provider, as typically occurs in TB and HIV treatment programs, may have on smoking cessation efficacy among these patients. Most importantly, there is a need to develop and test strategies for implementing evidence-based tobacco cessation interventions in the HIV setting, taking into account potential for sustainability and scale-up.

Here again, concepts and methods from implementation science can be instructive. The RE-AIM framework provides a structure for evaluating population based public health impact from multilevel interventions [97]. The framework emphasizes five dimensions on which programs should be evaluated: reach, efficacy, adoption, implementation, and maintenance. Hybrid effectiveness-implementation study designs also provide a method for evaluating both the effectiveness of a novel interventions while also taking account of their implementation and contextual factors [98]. And standardized reporting of details of the context in which an intervention is tested as well as the strategies used to implement it is important to inform scale-up and account for the role of local factors [99]. Additional attention is needed to support scale up of interventions beyond an individual clinic to a health system, which may require, for example, a train-the-trainer model to propagate a training strategy.

Nguyen and colleagues have studied the implementation of tobacco cessation treatment guidelines in Vietnam through the use of community health workers and health centers. They first studied barriers and facilitators to expanding the role of community health workers around tobacco cessation and then developed and tested an intervention for increased adherence to tobacco use treatment guidelines [100]. This work serves as a possible model for integrating tobacco cessation services into community HIV settings. As Theobald et al. have described, application of implementation science in global health requires particular attention to the local context, including contextualization of an intervention, addressing challenges relevant to the community, focusing on processes and outcomes, and being real world and real time focused [101]. Peek et al. also highlight the importance of substantive ongoing participation by stakeholders to produce research that is relevant [102], which is a critical consideration when taking an intervention developed in a HIC setting and studying it in an LMIC.

## 3. Conclusions

While tobacco use and tobacco-related cancers and other diseases disproportionately impact PLWH worldwide, there has been limited effort to date to address tobacco use in this setting. At the same time, as global investments have built healthcare delivery systems with the capacity to deliver HIV treatment consistently across diverse, low-resource environments, there is a tremendous opportunity to build on existing processes and infrastructure to deliver tobacco cessation interventions to PLWH. The HIV community has already demonstrated, for example, how local adaptations, such as task shifting and the use of community health workers, can enhance delivery of treatment to underserved groups. However, novel adaptations and strategies are likely needed for existing evidence-based tobacco cessation interventions to be implemented effectively for PLWH in LMICs. In order to begin to address these research gaps, the National Cancer Institute has recently released funding opportunities to support research on tobacco cessation and, more broadly, cancer prevention and control among PLWH in LMICs [103, 104].

While this paper has focused on opportunities for integrating tobacco cessation into the HIV treatment context, population-based tobacco control policies and programs remain critical to support tobacco cessation. Tobacco control policies, such as tax increases that raise the price of tobacco products and comprehensive smoke-free policies, and strong media campaigns also increase tobacco cessation at the population level [105]. Tobacco cessation interventions implemented through HIV clinics would be expected to have far greater impact when accompanied by strong, comprehensive tobacco control policies and programs.

The implementation of effective, evidence-based tobacco cessation interventions for PLWH in LMICs has potential to bring substantial benefits to health outcomes among PLWH, particularly in areas where the burden of both HIV and tobacco use is high. The United Nations Development Program has recently provided tools and guidance for countries to encourage incorporating tobacco control into their HIV and TB responses [106]. Further research grounded in implementation science as well as coordinated efforts across HIV and tobacco control programs have potential to address two major global health challenges and benefit PLWH and their families and communities.

## Figures and Tables

**Table 1:** Research needs along the implementation continuum for tobacco cessation among PLWH in LMICs.

	Preimplementation	Adaptation	Barriers and facilitators	Scale up and integration
Research targets	(i) Identify factors that influence likelihood of cessation in PLWH in LMICs (ii) Develop and test novel, targeted interventions for PLWH in LMICs (iii) Explore use of digital technologies for increasing reach or impact of interventions	(i) Adaptation of evidence-based interventions for PLWH from high-income settings to LMICs (ii) Adaptation of interventions previously tested in other challenging or low resource environments to PLWH in LMICs (iii) Adaptation of interventions to integrate tobacco cessation into the TB treatment context in LMICs for application in HIV clinics and treatment settings	(i) Understand the influence of local, contextual factors (such as tobacco use patterns) on tobacco cessation among PLWH in LMICs (ii) Understand individual, clinic, community, and policy barriers to tobacco cessation interventions for PLWH in LMICs (iii) Identify unique opportunities for intervention delivery (such as at the point of diagnosis)	(i) Evaluation of strategies to integrate and scale up tobacco cessation into existing HIV treatment settings (ii) Evaluation of implementation of guidelines for tobacco cessation in HIV treatment settings (iii) Understanding fidelity of implementation of tobacco cessation in the HIV context

Methods and frameworks	Application of social and behavioral theories and frameworks Studies of efficacy and effectiveness	Framework for reporting adaptations and modifications to evidence-based interventions (FRAME) Transferability (PIET-T model)	Exploration, preparation, implementation, sustainment (EPIS) Consolidated framework for implementation research (CFIR)	Reach effectiveness adoption implementation maintenance (RE-AIM) Hybrid study designs

## Data Availability

Review paper not including original data. Data related to the review is available on request.
